# Investigating the relationship between childhood sexual abuse, self-harm repetition and suicidal intent: mixed-methods study

**DOI:** 10.1192/bjo.2021.962

**Published:** 2021-07-08

**Authors:** Maria Isabela Troya, Grace Cully, Dorothy Leahy, Eugene Cassidy, Anvar Sadath, Sarah Nicholson, Ana Paula Ramos Costa, Íñigo Alberdi-Páramo, Anne Jeffers, Frances Shiely, Ella Arensman

**Affiliations:** School of Public Health, College of Medicine and Health, University College Cork, Ireland; and National Suicide Research Foundation, University College Cork, Ireland; School of Public Health, College of Medicine and Health, University College Cork, Ireland; and National Suicide Research Foundation, University College Cork, Ireland; School of Public Health, College of Medicine and Health, University College Cork, Ireland; and National Suicide Research Foundation, University College Cork, Ireland; Cork University Hospital Group, Liaison Psychiatry Service, Ireland; School of Public Health, College of Medicine and Health, University College Cork, Ireland; and National Suicide Research Foundation, University College Cork, Ireland; School of Public Health, College of Medicine and Health, University College Cork, Ireland; and National Suicide Research Foundation, University College Cork, Ireland; School of Public Health, College of Medicine and Health, University College Cork, Ireland; and National Suicide Research Foundation, University College Cork, Ireland; Instituto de Psiquiatría y Salud Mental, Hospital Clínico San Carlos, Spain; and Departamento de Medicina Legal, Psiquiatría y Patología, Universidad Complutense de Madrid, Spain; National Clinical Programme for the Assessment and Management of Patients presenting to the Emergency Department following Self-Harm, Office of the National Clinical Advisor and Group Lead – Mental Health, Dr. Steeven's Hospital, Ireland; School of Public Health, College of Medicine and Health, University College Cork, Ireland; School of Public Health, College of Medicine and Health, University College Cork, Ireland; National Suicide Research Foundation, University College Cork, Ireland; and Australian Institute for Suicide Research and Prevention, School of Applied Psychology, Griffith University, Australia

**Keywords:** Mixed-methods, suicide, self-harm, childhood sexual abuse, self-harm repetition

## Abstract

**Background:**

Research into the association between childhood sexual abuse (CSA) and self-harm repetition is limited.

**Aims:**

We aimed to examine the association between self-harm repetition, mental health conditions, suicidal intent and CSA experiences among people who frequently self-harm.

**Method:**

A mixed-methods study was conducted including consecutive patients aged ≥18 years, with five or more self-harm presentations, in three Irish hospitals. Information was extracted from psychiatric records and patients were invited to participate in a semi-structured interview. Data was collected and analysed with a mixed-methods, convergent parallel design. In tandem, the association between CSA and self-harm repetition, suicidal intent and mental health conditions was examined with logistic regression models and independent sample *t*-test, with psychiatric records data. Thematic analysis was conducted with interview data, to explore CSA experiences and self-harm repetition.

**Results:**

Between March 2016 and July 2019, information was obtained on 188 consecutive participants, with 36 participants completing an interview. CSA was recorded in 42% of the total sample and 72.2% of those interviewed. CSA was positively associated with self-harm repetition (odds ratio 6.26, 95% CI 3.94−9.94, *P* = 0.00). Three themes emerged when exploring participants’ CSA experiences: CSA as a precipitating factor for self-harm, secrecy of CSA accentuating shame, and loss experiences linked to CSA and self-harm.

**Conclusions:**

CSA was frequently reported among people who frequently self-harm, and associated with self-harm repetition. Identification of patients at risk of repetition is key for suicide prevention. This is an at-risk group with particular characteristics that must be considered; comprehensive patient histories can help inform and tailor treatment pathways.

Childhood sexual abuse (CSA) is a risk factor for several psychiatric conditions and behaviours, including self-harm.^1^ Although other factors can contribute to self-harm, evidence indicates that childhood maltreatment, including CSA, is directly associated with self-harm.^2^ Different aspects related to CSA, such as identity and relation to the perpetrator, and type and frequency of abuse, can further accentuate self-harm.^1,3^ Previous studies have assessed the evidence between CSA and self-harm, with findings ranging from small (*d* = 0.23)^4^ to medium (odds ratio 2.43−2.65)^2,5^ effect sizes for the association between CSA and self-harm. Other studies have established the association between CSA and self-harm within specific groups, such as adolescents,^6^ women,^7^ men^8^ and people who are imprisoned.^9^

Few studies have examined patients who frequently self-harm.^10,11^ This may be because of the overlap between people who frequently self-harm and people with emotionally unstable personality disorder or borderline personality disorder.^12^ Several theories support that self-harm among this clinical group is distinct, with varying functions of self-harm.^4,13^ Not everyone who engages in repeat self-harm will have such a clinical diagnosis; therefore, it is important to examine the clinical and sociodemographic characteristics of people who frequently self-harm. Specifically, few studies have addressed CSA among people who frequently self-harm.^14^

Research addressing suicidal intent among individuals who repeatedly self-harm is limited.^11^ However, evidence indicates that CSA is associated with experiencing higher levels of suicidality.^15^ The level of suicidal intent among people who frequently self-harm and its association with CSA warrant further exploration. There are ongoing knowledge gaps in relation to CSA among people who frequently self-harm. This research addressed whether people who frequently self-harm with previous CSA history have increased self-harm repetition, suicidal intent and/or mental health conditions. We examined CSA among people who frequently self-harm, using a mixed-methods approach to obtain a more comprehensive view of the experiences of CSA and clinical outcomes. Specific objectives were to describe the clinical characteristics of a consecutive sample of people who frequently self-harm; examine the relationship between CSA and selected sociodemographic and clinical variables; examine the association between CSA and frequent self-harm repetition, suicidal intent and mental health conditions; and examine the experiences of CSA among people who frequently self-harm.

## Method

### Setting and design

This is a mixed-methods study with convergent parallel design in which quantitative and qualitative data were collected and analysed simultaneously.^16^ The study was conducted in three emergency departments in the south (Cork) and west (Limerick) of Ireland, where consecutive self-harm presentations were identified for the quantitative study. Patients were invited to take part in a semi-structured interview for the qualitative study. Data were obtained from March 2016 to July 2019 (inclusive).

### Sample and procedure

Inclusion criteria were people who frequently self-harm, were aged ≥18 years and presented with self-harm to one of the three participating hospitals. Frequent self-harm was defined as five or more previous self-harm presentations to hospital emergency departments, including the index presentation. Self-harm was defined as ‘an act with non-fatal outcome where an individual deliberately initiates a non-habitual behaviour that without intervention from others will cause self-harm’.^17^ This included both acts with and without the intention to die. For one of the variables analysed, we included a comparison group of patients with high risk self-harm, who were aged ≥18 years and presented to one of the three participating hospitals with self-harm of high lethality and/or high levels of suicidal intent and a history of fewer than five self-harm presentations. High risk self-harm was defined as in previous studies.^18,19^ Participants were recruited regardless of whether they had already a previous presentation.

Psychiatric staff from the three participating hospitals were informed of the study by members of the research team (G.C., S.N., D.L. and E.A.), and provided with information sheets for inclusion/exclusion criteria for eligible participants. Psychiatric staff applied inclusion criteria after identifying consecutive cases of self-harm. Researchers were provided with access to psychiatric files. Information about psychiatric history, including previous self-harm, medical conditions, CSA and suicidal intent, was obtained from psychiatric records (Supplementary File 1 available at https://doi.org/10.1192/bjo.2021.962). Suicidal intent, routinely assessed by staff in all self-harm presentations, was assessed with the eight objective items of the Beck Suicide Intent Scale (SIS), which has good internal consistency reliability in the current sample (Cronbach's *α* = 0.959).^20^ Previous research conducted in emergency department settings used the eight objective items of the Beck SIS to measure suicidal intent as part of routine assessment, measuring objective circumstances of self-harm.^21^ The eight objective items of the Beck SIS were used in this study because of the possibility of this being integrated into routine assessments and measuring suicidal intent. All data from psychiatric records (including any medical diagnoses) were as recorded by the psychiatric nurse or doctor conducting patient assessments. If a patient was identified more than once during the data collection period, the index presentation was considered.

Following an assessment of the patient by psychiatric staff, the study was explained to patients to verify if they were interested in participating in the semi-structured interview. Patients were excluded if they were physically (e.g. unconscious and/or induced coma) or mentally (e.g. unable to provide informed consent because of a lack of capacity) unable to take part, as identified by psychiatric staff. Participants were not included if they were unable to provide informed consent; for example, if they were mentally unable to comprehend the research, or it was deemed inappropriate by the psychiatric staff because the patient was expressing aggressive behaviour with possible risks for the researcher.

After patient agreement, a researcher provided an introduction of the research, using an invitation letter and information leaflet via post or in person while on the hospital premises. When patients were perceived well enough to take part and consented, interviews were held in a private room in the hospital. Alternatively, researchers contacted participants via telephone to see if they were interested in participating in the interview study. If individuals agreed, their preferred time and venue (home, university premises, other) were prioritised when scheduling the interview. A total of 31 of the 36 interviews were audio-recorded (time range of 69–258 min); the remaining interviews were not audio-recorded, per participant request.

Supplementary File 2 provides a list of the information collected via the semi-structured interviews, which includes demographic information, medical history and engagement with healthcare services, with a combination of open-ended questions and instruments used to measure psychosocial variables. Open-ended questions were included relating to the index self-harm, events leading to self-harm and self-harm repetition, among others.

### Ethics

The authors assert that all procedures contributing to this work comply with the ethical standards of the relevant national and institutional committees on human experimentation and with the Helsinki Declaration of 1975, as revised in 2008. The study was approved by the Clinical Research Ethics Committee of the Cork Teaching Hospital (reference number EMC 4 (2) 12/04/16) and the Health Service Executive Mid-Western Regional Hospital Research Ethics Committee (reference number REC 018/6). Ethical approval was obtained before the implementation of the General Data Protection Regulation (GDPR).^22^ Following the implementation of GDPR, information leaflets and consent forms were updated to comply with GDPR and subsequently approved by the ethics committees. Given the study population and perceived potential of identifying participants at risk of suicide, researchers who conducted interviews assessed suicide risk throughout the interview. With the agreement of the participant, a follow-up contact was established via telephone 10 days after interviews, to ensure that any needs or potential effects from the interview were identified, monitored and counselled.

### Analysis

As per the convergent mixed-methods design, analysis from the quantitative and qualitative data happened concurrently.^16^

#### Quantitative data analysis

Statistical analysis was performed with the statistical package SPSS for Windows, version 26. Patient files were excluded from the analysis when there was no assessment on file for the self-harm presentation. Individuals presenting witht high risk self-harm who had five or more self-harm presentations on record were included in the frequent self-harm group only, and those who had fewer than five self-harm presentations were included in the high riskself-harm group. Descriptive statistics were used to report demographics and psychiatric characteristics of participants. Cross-tabulation and Pearson *χ*^2^-test were used to explore the relationship of selected sociodemographic (gender, marital status, employment) and clinical variables (personality disorder, physical illness) between people who frequently self-harm with and without a history of CSA. Significance levels were set at *P* < 0.05. When there was missing data in one of the variables included in the descriptive analysis, these values were excluded from the percentage (missing values ranging from 0 to 58). The association between CSA and suicidal intent among the frequent self-harm group was tested with an independent sample *t*-test, where SIS scores were treated as a continuous and dependent variable and CSA was treated as grouping variables with two categories (yes/no) (95% confidence intervals). The association between CSA and self-harm repetition, and mental/physical illness was analysed with binomial logistic regression (95% confidence intervals). Experiencing CSA and self-harm repetition was compared by using the frequent self-harm and high risk self-harm groups. A sample size of 506 people who self-harm (318 people with high risk self-harm and 188 people who frequently self-harm), of whom 60% had no history of CSA and 40% had a history of CSA, provided 85% power at a 5% level of statistical significance to detect an odds ratio of 1.75 with logistic regression.^23^ All other analysis was done with people who frequently self-harm exclusively. All categorical variables were dummy coded (1 indicating yes and 0 indicating no diagnoses of mental/physical illness). A sample size of 188 people who frequently self-harm, of whom 60% had no history of CSA and 40% had a history of CSA, provided 86% power at a 5% level of statistical significance to detect an odds ratio of 2.5 with logistic regression.^23^ For the logistic regression analyses, we performed multiple imputation methods for variables with missing data of ≥15%. Logistic regression analyses are reported with and without missing values analysis, and pooled estimates are presented.

#### Qualitative data analysis

Qualitative analyses were conducted by a four-person multidisciplinary team, using thematic analysis. Braun et al's^24^ approach was used to analyse interviews. Author M.I.T. led the analysis process, with at least two co-authors contributing to the coding of each interview and emerging themes. All transcripts were coded by at least two of the authors, and discussed among co-authors leading the qualitative analysis (M.I.T., A.S., S.N. and E.A.). Emerging themes were reviewed, discussed until consensus reached and refined collaboratively by authors M.I.T., A.S., S.N. and E.A. Thematic analysis allowed the qualitative and quantitative data analyses to be integrated, and complemented the mixed-methods approach. Data analysis software manager QSR International NVivo (Australia) for Windows version 12 (https://www.qsrinternational.com/nvivo-qualitative-data-analysis-software/home) was used to assist in the analysis of the qualitative data-set.

## Results

Between March 2016 and July 2019, 191 consecutive people who frequently self-harm were identified; nine patients were excluded from the analysis because there were no file assessments. Six files from the high risk self-harm group were moved to the frequent self-harm group because they had five or more self-harm presentations; thus, 188 files were included in the frequent self-harm group. Of the 188 identified files, 36 participants consented to the interview study (Supplementary File 3 summarises reasons for not participating in the interview study). In the comparison group, 345 consecutive patients with high risk self-harm were identified and 318 were included in the analysis, after removing files where no assessment was provided and removing the six identified patients with high risk self-harm with five or more self-harm presentations. Results are presented as consecutive psychiatric records for the quantitative analysis (*n* = 188) and interview data for the qualitative analysis (*n* = 36).

### Consecutive psychiatric records: quantitative data

The mean age of people in the frequent self-harm group was 37.38 (s.d. 12.92), and most were women (60.1%). Intentional drug overdose (61.2%) was the most reported method, with 55.7% of participants having overdosed on their prescribed medication. Seventy-nine of the 188 people in the frequent self-harm group (42.0%) reported a history of CSA. Most (97.8%) had a recorded mental health condition, with personality disorders being the most common (45.4%). Over three-quarters (76.2%) had more than one mental health condition. More than half (55.4%) had a recorded physical illness. Over a third (37.8%) reported both physical and mental health comorbidities. Table 1 summarises the sociodemographic and clinical characteristics of the frequent self-harm group.

**Table 1:** Sociodemographic and medical characteristics of people who frequently self-harm in relation to reported CSA (*N* = 188)

	People who frequently self-harm with history of CSA (*n* = 79)	People who frequently self-harm with no history of CSA (*n* = 109)	All people who frequently self-harm (*n* = 188)
Gender			
Male	25 (68.4%)	50 (45.9%)	75 (39.9%)
Female	54 (31.6%)	59 (54.1%)	113 (60.1%)
Age, years, mean	36.22 (s.d. 11.405)	38.22 (s.d. 13.903)	37.38 (s.d. 12.92)
Relationship status			
Single	40 (50.7%)	65 (59.6%)	105 (56.1%)
Married/cohabiting/relationship	29 (36.7%)	27 (24.8%)	56 (29.7%)
Widowed/divorced/separated	8 (10.1%)	16 (14.7%)	24 (12.7%)
Not reported/unknown	2 (2.5%)	1 (0.9%)	3 (1.5%)
Living arrangements			
Alone	19 (24.1%)	27 (24.8%)	46 (24.5%)
With family of origin/spouse/children	43 (54.4%)	63 (57.8%)	106 (56.4%)
Others (e.g. friends)	13 (16.5%)	14 (12.8%)	27 (14.3%)
Not reported/unknown	4 (5.0%)	5 (4.6%)	9 (4.8%)
Employment status			
Employed	11 (13.9%)	16 (14.7%)	26 (13.8%)
Unemployed	40 (50.7%)	58 (53.2%)	98 (52.1%)
Long-term disability	13 (16.5%)	12 (11.0%)	25 (13.3%)
Full-time student	8 (10.1%)	8 (7.3%)	16 (8.5%)
Other (retired/housewife, etc.)	3 (3.8%)	3 (2.8%)	7 (3.7%)
Not reported/unknown	4 (5.0%)	12 (11.0%)	16 (8.6%)
Current self-harm method (if multi-method, main method recorded)			
Drug overdose	47 (59.5%)	68 (62.4%)	115 (61.2%)
Hanging/strangulation/suffocation	4 (5.0%)	9 (8.3%)	13 (6.9%)
Cutting	23 (29.2%)	26 (23.8%)	49 (26.1%)
Submersion (drowning)	2 (2.5%)	3 (2.8%)	5 (2.7%)
Other (fire/crashing vehicle)	3 (3.8%)	3 (2.8%)	6 (3.1%)
Multiple methods of self-harm			
Yes	5 (6.3%)	11 (10.1%)	16 (8.5%)
No	73 (92.4%)	96 (88.1%)	169 (89.9%)
Not reported/unknown	1 (1.3%)	2 (1.8%)	3 (1.60%)
Alcohol consumed at the time of self-harm			
Yes	35 (44.3%)	55 (50.5%)	90 (47.9%)
No	34 (43.0%)	40 (36.7%)	74 (39.3%)
Not reported/unknown	10 (12.7%)	14 (12.8%)	24 (12.8%)
Suicidal intent			
Beck SIS score, mean	3.71 (s.d. 2.50)	3.61 (s.d. 2.27)	3.65 (s.d. 2.36)
Recorded mental health conditions			
Yes	76 (97.4%)	105 (97.2%)	181 (97.8%)
No	2 (2.6%)	3 (2.8%)	4 (2.2%)
More than one recorded mental health condition^a^			
Yes	58 (75.3%)	83 (76.9%)	141 (76.2%)
No	19 (24.7%)	25 (23.1%)	44 (23.8%)
Type of mental health condition^b,c^			
Depressive disorder			
Yes	16 (25.9%)	41 (45.1%)	57 (37.2%)
No	46 (74.1%)	50 (54.9%)	96 (62.8%)
Bipolar disorder			
Yes	8 (12.1%)	6 (7.2%)	14 (9.4%)
No	58 (87.9%)	77 (92.8%)	135 (90.6%)
Personality disorder			
Yes	39 (58.2%)	30 (35.3%)	69 (45.4%)
No	28 (41.8%)	55 (64.7%)	83 (54.60%)
Psychosis/schizophrenia			
Yes	2 (3.0%)	7 (8.1%)	9 (5.9%)
No	65 (97.0%)	80 (91.9%)	145 (94.1%)
Anxiety disorders			
Yes	13 (20.9%)	26 (29.5%)	39 (26.0%)
No	49 (79.1%)	62 (70.5%)	111 (74.0%)
Eating disorders			
Yes	3 (4.7%)	3 (3.6%)	6 (4.1%)
No	61 (95.3%)	80 (96.4%)	141 (95.9%)
Alcohol dependence			
Yes	21 (31.8%)	17 (18.5%)	38 (24.1%)
No	45 (68.2%)	75 (81.5%)	120 (75.9%)
Drug dependence			
Yes	12 (17.6%)	14 (14.7%)	26 (15.9%)
No	56 (82.4%)	81 (85.3%)	137 (84.1%)
Prescribed psychiatric medication			
Yes	63 (86.3%)	81 (83.5%)	144 (84.7%)
No	10 (13.7%)	16 (16.5%)	26 (15.3%)
Prescribed medication for physical illness			
Yes	18 (37.5%)	34 (49.3%)	52 (44.6%)
No	30 (62.5%)	35 (50.7)	65 (55.6%)
Recorded physical illness^d^			
Yes	27 (54.0%)	45 (56.9%)	72 (55.4%)
No	23 (46.0%)	34 (43.1%)	58 (44.6%)
Type of physical illness			
Physical pain	4 (5.1%)	9 (8.3%)	13 (6.9%)
Epilepsy/seizures	2 (2.5%)	11 (10.1%)	13 (6.9%)
Asthma	5 (6.3%)	5 (4.6%)	10 (5.3%)
Thyroid problems	2 (2.5%)	6 (5.5%)	8 (4.2%)
Heart disease	4 (5.1%)	4 (3.7%)	8 (4.2%)
High cholesterol	1 (1.3%)	6 (5.5%)	7 (3.7%)
Gastrointestinal problems	3 (3.8%)	5 (4.6%)	8 (4.2%)

All data are given as *n* (%), unless otherwise stated. CSA, childhood sexual abuse; SIS, Suicide Intent Scale.

a. Patients could have more than one mental health condition recorded.

b. Recorded diagnoses by psychiatric nurse or doctor who made the assessment of the patient and recorded in the file review.

c. Missing values for recorded mental health condition ranged from 0 to 41. Data presented on mental illness excludes unknown values.

d. Missing values for recorded physical illness ranged from 0 to 58. Data presented on physical illness excludes unknown values.

#### Relationship between CSA and selected variables among people who frequently self-harm

We examined sociodemographic and clinical variables among people who frequently self-harm with and without a history of CSA (see Table 2). There was a significant gender difference, with more women having CSA experiences than men (*χ*^2^ *=* 3.86, *P* *=* 0.05*).* Personality disorder prevailed among participants with a history of CSA (*χ*^2^ *=* 7.94, *P* *=* 0.01).

**Table 2:** Relationship between CSA and selected sociodemographic and clinical variables

Variable ^a^	Dual category	CSA							
		No		Yes		*χ*^2^	*P*-value	Φ	*P*-value
		Count	% within variable	Count	% within variable				
Gender	Male	50	66.7%	25	33.3%	3.86	0.05	0.14	0.04
	Female	59	52.2%	54	47.8%				
Marital status	Single	81	62.3%	49	37.7%	2.78	0.14	0.12	0.09
	Married/relationship	27	49.1%	28	50.9%				
Employment	Employed	15	57.7%	11	42.3%	0.21	0.89	0.01	0.88
	Unemployed	82	56.2%	64	43.8%				
Personality disorder	No	55	66.3%	28	33.7%	7.94	0.01	0.22	0.01
	Yes	30	43.5%	39	56.5%				
Physical illness	No	34	58.6%	24	41.4%	0.20	0.65	0.03	0.65
	Yes	45	62.5%	27	37.5%				

All variables were binary. For marital status: single category included separated, divorced and widowed. Married category included in a short- or long-term relationship and cohabiting. For employment status: employed category included paid and self-employed. Unemployed category included full-time student, disability, retired and housewife/husband. CSA, childhood sexual abuse.

a. Missing values ranged from 3 to 58.

#### Association between CSA and self-harm repetition, suicidal intent, and mental and physical illness

Those with a history of CSA were 6.26 times more likely to experience self-harm repetition (five or more self-harm presentations) (95% CI 3.94−9.94, *P* = 0.00) than those with no history of CSA. In terms of suicidal intent, there was no association between people who frequently self-harm with or without a history of CSA and levels of intent. No association was found between CSA and personality disorder or CSA and mental/physical comorbidities (see Tables 3 and 4).

**Table 3:** History of childhood sexual abuse as a predictor of self-harm repetition and mental and physical illness

Tested variable	*β*	s.e.	Wald's *χ*^2^	d.f.	Odds ratio	95% CI for Exp(B)		*P*-value
						Lower	Upper	
Five or more self-harm presentations	1.83	0.24	60.45	1	6.26	3.94	9.94	0.00
Personality disorder								
Original data	0.94	0.34	7.79	1	2.55	1.32	4.93	0.01
Missing data analysis	0.73	0.38			2.08	0.96	4.47	0.06
Mental/physical health comorbidity	−0.27	0.31	0.74	1	0.39	0.42	1.40	0.78

Missing values of *n* = 36 for personality disorder and *n* = 3 for mental/physical comorbidity. Exp(B), exponentiation of the B coefficient.

**Table 4:** Association of CSA and suicidal intent among people who frequently self-harm

CSA	Frequency ^a^	Mean (s.d.)	*T*	Significance
Yes	68	3.71 (2.50)	−0.26	0.94
No	92	3.61 (2.27)		

Dependent variable was the Beck's Suicide Intent Scale total score. CSA, childhood sexual abuse.

a. Missing values *n* = 28.

### Interview data

Of the 188 identified people who frequently self-harm, 36 participated in the interview study, 66.6% of whom were women. There were no significant differences between individuals participating in the interview study and non-participants with respect to age, gender and marital status. Self-harm methods for interviewed participants were drug overdose (75%), cutting/stabbing (19.4%) and attempted hanging (5.6%). CSA was reported by 72.2% of interviewed participants. Sexual assault, defined as any sexual assault experienced at ≥17 years, was reported by 41.6%. In terms of mental health conditions, personality disorder (52.7%), depression (41.6%), anxiety disorder (33.3%) bipolar disorder (25%) and schizophrenia (16.6%) were the most reported conditions. Alcohol misuse/dependence was reported among 52.7% participants, and 41.6% reported drug misuse/dependence. The most common physical conditions were orthopaedic illness (22.2%), asthma (22.2%), gut disease (19.4%) and metabolic disease (19.4%) (see Table 5).

**Table 5:** Characteristics relating to medical diagnoses of people who frequently self-harm

Medical diagnoses ^a^	Interview data (*n* = 36)	Psychiatric records (*N* = 188)
Mental health condition	35 (97.2%)	181 (97.8%)
Physical illness ^b^	26 (72.2%)	72 (55.4%)
Comorbidities	25 (69.4%)	71 (37.8%)
Chronic physical illness	19 (52.7%)	Not provided
Physical pain ^c^	26 (72.2%)	13 (6.9%)
Reduced physical capabilities	21 (58.3%)	Not provided

a. Medical diagnoses were recorded as reported by participants.

b. Missing values for recorded physical illness from psychiatric records ranged from 0 to 58. Data presented on physical illness excludes unknown values.

c. Self-reported physical pain experienced in the past 12 months.

#### Qualitative analysis

Three main themes emerged from the data-set when exploring experiences of CSA and self-harm: CSA as a precipitating factor for self-harm throughout different stages of the life course, secrecy of CSA accentuating feelings of shame, and experiences of loss being linked to CSA, self-harm and limited coping methods.

#### Theme 1: CSA as a precipitating factor for self-harm throughout different stages of the life course

Most participants described the start of their self-harm as a response to traumatic abuse experiences. Self-harm was described as a way of coping with trauma, and this form of coping persisted in later life. Participants associated the traumatic experiences of CSA and self-harm, perceiving them as interconnected:

‘I just felt the need to harm myself. She [doctor] wrote that I had … not come to terms with it [CSA] yet and … my inappropriate behaviours were my way of dealing with it’ MR 226 (female).

‘I get constant flashbacks of the abuse and my perception of healthy relationships isn't very accurate or healthy. And probably one of the main reasons that I am still in and out hospital trying to kill myself’ MR 250 (female).

For most participants, CSA experiences affected their romantic relationships. Most reported their abuse experiences from close family members. As a result, participants described having trust issues/difficulties with family members. Difficulties in trusting family members continued to have an effect on participants’ future relationships with family members and new romantic relationships:

‘The sexual abuse that would have been major. That's definitely shaped my life. I think, definitely things aren't right for me, in a sexual way. Because …, I find … usually, if I had sex with somebody afterwards I feel awful’ MR 241 (female).

‘I froze up sexually with my husband, I used to freeze whenever he came to bed but obviously it was PTSD [post-traumatic stress disorder], you're re-living something, I didn't know it then … When we'd be sleeping and if my husband were to touch me I'd just scream “get off me, get off me,” it's the night terrors’ MR 102 (female).

‘Being sexually abused has taken a lot from me. Another reason my ex broke up with me is ‘cause I am on a very different place sexually. He couldn't really understand being sexually abused has taken a lot of joy away in my life’ MR 232 (female).

#### Theme 2: secrecy of CSA accentuating feelings of shame

CSA was often kept a secret between the perpetrator and participant for several years because of a fear of the perpetrator. Therefore, self-harm that started because of CSA was also kept hidden by participants. As summarised in the following quote, keeping the CSA experience a secret often meant that participants were left to deal with the traumatic experience on their own:

‘I was 13 when it happened to me. I wasn't told what was right or wrong. I didn't say that to my sister, didn't say that to my father, just kept it to myself. I never sought help, I just buried my head inside the pint to drown my sorrows’ MR 104 (male).

Feelings of having to hide their CSA experiences and self-harm often meant participants felt shame as a result of both experiences. Furthermore, even when sharing the CSA and self-harm experiences with close family members, shame and stigma was reported. In some cases, participants felt unfairly treated when they accused a family member of CSA:

‘Some people that get abused get support from their families. My sisters said I brought shame to the family by telling and having him charged, all my fault everything was my fault so they blame me for bringing shame to the family’ MR 380 (female).

‘When it [abuse] came out, my mam's family were split. It's your own fault, if you hadn't told, you would still be going to these [family] parties …. ’ MR 292 (female).

‘I got more abuse from my own family when I told them what happened to me, it was like I wasn't a victim’ MR 104 (male).

#### Theme 3: experiences of loss being linked to CSA, self-harm and limited coping methods

After several years of dealing with the traumatic CSA experiences, some participants disclosed the abuse to close family members. Some participants reported losing social support from family members, given that they were often the perpetrators or close to the perpetrators. Therefore, participants were estranged from their family, losing social support:

‘I cut all contact with them; I found it easier not to talk to them and not to confront what's going on in my head in case I act impulsively’ MR 255 (male).

Other loss experiences were also reported: bereavement, loss of custody of children and loss of physical function. Participants stated that loss was one of the main factors leading to self-harm. Respondents described limited coping methods to deal with life adversities such as loss or CSA, and therefore they often relayed on self-harm to deal with such adversities. Most participants had health comorbidities. Loss of physical function because of physical conditions often meant that participants lost their jobs, resulting in a further sense of loss of meaning. Financial strain caused by job loss as a result of ill health exacerbated participants’ worries:

‘What influences me harming myself is just the abuse when I was a young fellow and losing my girlfriend to suicide and started drinking then for 16 years every day of the week’ MR 223 (male).

## Discussion

### Main findings

This is one of few studies addressing CSA among patients who self-harm with five or more previous self-harm episodes. A key finding was that those with a history of CSA were 6.26 times more likely to experience self-harm repetition (five or more self-harm presentations) compared with those without a history of CSA. Consistent with the quantitative results, qualitative findings showed that a history of CSA was a precipitating factor for self-harm throughout different stages of the life course, and represented a reason for continued engagement in self-harm. Although the findings regarding the associations between personality disorder and CSA history are not conclusive, further exploration is needed. Suicidal intent was relatively low among people who frequently self-harm, and no significant association was found between CSA and suicidal intent.

In our cohort, people who frequently self-harm were predominantly women, middle-aged, single, living accompanied and unemployed. Alcohol was often consumed as part of the self-harm act, and intentional drug overdose was the most common method. Female gender and recorded personality disorders were higher in those who frequently self-harm who had experienced CSA. Among the frequent self-harm group, record of medical conditions was high. However, certain information was not provided in psychiatric files, including chronic physical illness and reduced physical capabilities. Interview data captured such missing information, and found high levels of chronic physical illness, reduced physical capabilities and physical pain in the past year.

Qualitative data revealed that loss experiences and limited coping mechanisms were often linked to CSA and self-harm. Furthermore, secrecy of CSA accentuated feelings of shame among people who frequently self-harm. These are important aspects to consider when assessing a patient presenting with self-harm, as secrecy can cause shame and limit help-seeking. We observed a higher proportion of CSA reported in the interview study when comparted with psychiatric assessment data, suggesting that participants were not inclined to immediately disclose this information during their assessment.

### Comparison with previous research

Previous studies among the general population in Ireland have found that 16% of men and 20% of women report a history of CSA.^25^ Evidence examining self-harm and CSA is limited among individuals engaging in repeat self-harm, both in Ireland and internationally. One study sought to examine CSA with other types of abuse experienced in childhood, among a population of patients admitted to hospital following self-harm.^26^ The respective study found that CSA was associated with repeated suicidal behaviour; however, their prevalence of CSA (33%) was significantly lower than the one found in our study. The findings of our study not only add to this evidence, but also accentuate the future risk of repeated self-harm among individuals who have experienced CSA.

Furthermore, this study adds further understanding of the increased risk of comorbid health conditions in people who frequently self-harm. Previous research has shown how comorbid mental and physical health conditions, including, but not limited to, depression, bipolar disorder, personality disorders, cancer, sleep disorders and heart disease, are risk factors for self-harm and suicide.^27–34^ Comorbid physical health conditions have been found to be associated with increased self-harm, particularly among patients with chronic or long-standing conditions, such as cancer and heart disease.^29,31^ Our findings show that prevalence of both mental and physical health conditions is high among people who frequently self-harm. Therefore, during clinical assessments and when considering treatment options (e.g. access to means), particular attention should be given regarding the implications of physical and mental ill health of patients who self-harm. However, our study found no association between CSA and mental/physical health comorbidity. This suggests that having a history of CSA primarily affects the future mental health of people who frequently self-harm. However, previous studies have found long-standing effects of CSA on physical and mental health.^35^ Furthermore, previous studies have shown that CSA can affect somatic symptoms, both related to physical and mental health conditions, such as somatisation disorder or psychogenic pain.^36–38^ Given the included variables in our study, we were unable to assess if the recorded medical conditions were psychosomatic, but this would be important to consider in future studies and clinical practice.

Our research found relatively low levels of suicidal intent among people who frequently self-harm, which is consistent with previous research.^11^ However, previous research has found that patients with a history of CSA report high suicidal intent.^15^ Furthermore, our study found no significant association between CSA and suicidal intent. However, suicidal intent was measured through the eight-item objective scale recorded by the psychiatric staff conducting the assessment. Some patients may have had higher levels of suicidal intent at the time of assessment. Previous studies have reported how measuring suicidal intent is complex and, when done in first-contact settings, can be imprecise because of the emotional state of patients, attitudes of healthcare professionals and ambiguity of patients’ responses.^20,39,40^ Therefore, findings of low suicidal intent among the people who frequently self-harm warrant caution when interpreting results. Although some research proposes that self-harm can be dichotomised in terms of suicidal intent (e.g. non-suicidal self-injury versus attempted suicide) according to frequency of self-harm or methods used,^41^ the outcomes of the present study are in line with evidence indicating that suicidal intent is a fluid concept that cannot be dichotomised.^42^ Suicidal intent is complex and sometimes cannot be fully ascertained in first-contact settings.

Qualitative findings show the long-standing effect CSA had on participants, in particular when the perpetrators were family members. This is consistent with previous research that found that a perpetrator's identity is linked with future self-harm, with victims of family perpetrators being at highest risk.^1,3^ The qualitative findings add to this by showing the long-standing effects of CSA from family perpetrators influencing participants’ future relationships. Our findings are consistent with previous studies reporting that previous traumatic experiences, including CSA, are linked with self-harm in later life.^43–45^

### Strengths and limitations

The mixed-methods design of this study allowed for in-depth investigation of relevant quantitative variables and qualitative data. The combination of quantitative and qualitative data complemented and strengthened key findings from this research (e.g. effect of CSA on self-harm repetition). Data collected from the interviews allowed in-depth comprehension of CSA experiences. Reporting on consecutive cases of people who frequently self-harm who presented to three major hospitals, our study comprehensively covered consecutive emergency department self-harm presentations for this subgroup. Nevertheless, our findings should be interpreted in consideration of the following limitations. First, the uptake to the interview study was relatively low (19%), albeit in line with previous studies involving people who frequently self-harm.^46^ Second, we present data on a specific population: individuals who present to the hospital with self-harm. Our findings are therefore only applicable to a selected group and not representative to self-harm occurring in the community. Furthermore, eligible participants had to have capacity to consent, being both physically and mentally able to take part in this research. This meant that certain groups of patients could not be considered for the study (e.g. patients who were unconscious or in induced coma because of the severity of the self-harm act), and valuable lived experience was missed from this group. Third, our findings report retrospective data, and must be considered in light of this design. Fourth, as our study reports on information collected as part of routine psychiatric assessments, there was a considerable amount of missing data; in particular, physical illness (*n* = 58) and personality disorders (*n* = 36) had the highest numbers of missing data. Therefore, our findings should be interpreted with caution. However, we performed a missing data analysis to adjust results of variables with ≥15% of missing data. Lastly, when examining the association between CSA and self-harm repetition,patients withhigh risk self-harm were the comparison group. Despite these patients having fewer than five self-harm presentations, the characteristics and clinical profile of patients withhigh risk self-harm mean that the differences between people who frequently self-harm and patients with high risk self-harm are more complex than mere self-harm repetition, and should therefore be considered when interpreting results.^18^ For instance, patients with high risk self-harm may die by suicide at a higher or quicker rate than people who frequently self-harm, which could lead to lower emergency department presentations. However, overlap does exist among patients with high risk self-harm and people who frequently self-harm, with previous studies reporting over half of patients withhigh risk self-harm had previous history of self-harm.^18^ Future research could examine the overlapping characteristics of the two groups.

### Clinical and research implications

Identification of individuals at risk of self-harm repetition and future suicide is key for suicide prevention. People who frequently self-harm are an at-risk group, with high prevalence of CSA, record of mental and physical conditions and access to prescribed medications because of their medical conditions. The identification of such characteristics is essential, and needs to be considered when conducting biopsychosocial assessments. Furthermore, such patient history should inform and tailor the recommended treatment for these patients.

Inclusion of patients with lived experience (e.g. through qualitative research) can provide unique insights, depth and breadth to data that is often missed by purely quantitative research.^47^ Future research into suicide prevention may benefit from the lived experience of people with previous self-harm history, as conducted in our mixed-methods research.

Previous research has already demonstrated that biopsychosocial assessments are key for self-harm prevention, as provision of assessments are associated with reduced repetition.^48–52^ However, evidence shows that both structured and unstructured risk assessment tools of future risk following self-harm are not accurate enough to be clinically useful.^53,54^ Semi-structured assessment templates can improve the completeness of information recorded, although whether this improves outcomes is still unclear.^55,56^ The provision of broader semi-structured biopsychosocial assessments is essential when conducting clinical assessments in patients who self-harm. Among people who frequently self-harm, conducting thorough biopsychosocial assessments at each presentation is important, given that patients’ circumstances may have deteriorated from the previous self-harm episode, and new information may emerge.^47^ Findings from our study underscore the importance of conducting thorough biopsychosocial assessments that include not only medical history, but also wider environmental, social and personal history. This should be applied among all people who self-harm, and particularly those who frequently self-harm, as repeated hospital presentations can often result in omissions and ill-informed assumptions.^17^

## Data Availability

The data that support the findings of this study are available on request from the corresponding author, M.I.T. The data are not publicly available due to the nature of the research containing sensitive information that could compromise the privacy of research participants.

## Supplementary material

For supplementary material accompanying this paper visit http://dx.doi.org/10.1192/bjo.2021.962. click here to view supplementary material
