# Supplementary material for: Investigating the relationship between childhood sexual abuse, self-harm repetition and suicidal intent: mixed-methods study
Source: BJPsych Open. 2021 Jul 8;7(4):e125. doi: 10.1192/bjo.2021.962 (PMC8281309; doi:10.1192/bjo.2021.962)
Supplement: Supplementary file 1 [file S2056472421009625sup001.zip › S2056472421009625sup009.docx]

## Appendix 1. Information collected in psychiatric records data

| **Name** | **Description** |
| --- | --- |
| Participant Type | Major Repeater or High-Risk |
| Assessment on file | Assessment on file available yes/no |
| Hospital | Hospital: CUH/Mercy/Limerick |
| Date and time of presentation | Date and time of self-harm presentation |
| Mode of presentation | Mode of presentation to hospital |
| Date of Birth | Date of birth of self-harm patient |
| Age | Age at time of presentation |
| Sex |  |
| Nationality |  |
| Marital Status | Marital Status |
| Accommodation | Accommodation |
| Living Arrangements | Living Arrangements |
| Number of children | Number of children |
| Employment Status | Employment Status |
| Current profession | Current profession if employed |
| Sector of employment | Sector of employment |
| In receipt of social welfare | In receipt of social welfare yes/no |
| Orphanage or Foster care | History of Residence in Orphanage or Foster care |
| Forensic history | History of criminal activity |
| Method of self-harm | Method of self-harm used |
| Multimethod | Multiple methods of self-harm |
| Name of medication | If self-poisoning, substance prescription name |
| Medication self-poisoning | If self-poisoning where did the substance come from |
| Alcohol | Alcohol consumed as part of the act |
| Beck Suicidal Intent Scale 8-items objective section | Questions: isolation, timing, precautions, acting to get help, final acts, active preparation, suicide note, overt communication |
| Proximal Factors | Factors influencing self-harm: unemployment, financial difficulties, legal troubles, disruption of relationship, interpersonal conflict, addiction |
| Victim of assault | Victim of violent or sexual assault at some time in the past |
| Vehicle collision | Involved in significant vehicle collision at some time in the past |
| Victim of abuse | Victim of abuse (sexual, physical, domestic, neglect) |
| Witness of violent crime | Witness Of Violent Crime at some time in the past |
| Witnessed sudden death | Directly witnessed sudden death at some time in the past |
| Severe bullying or torture | Severe bullying, torture |
| War, terrorism or natural disaster | Directly experienced war, terrorism, natural disaster in the past |
| Unexpected death | Experienced the sudden, unexpected death of a loved one |
| Suicide of loved one | Completed suicide of loved one |
| Bereavement of loved one | Bereavement of loved one |
| Family History | Family history of mental illness, alcohol abuse, substance abuse |
| Prior Self-harm | Prior self-harm |
| Mental health diagnosis | Diagnosis of mental health disorder |
| Previous mental health treatment | Inpatient, outpatient, addiction services, private counselling, GP |
| Psychiatric medication | Taking psychiatric medication |
| Physical illness diagnosis | Diagnosed physical illness or physical symptoms |
| Physical Medication | Taking medication for physical health condition |
| After index self-harm treatment | After index self-harm episode, admission to Psychiatric unit or hospital, outpatient, or community based mental health services, GP, counselling, social work or change in psychiatric medication |
