# Supplementary material for: Investigating the relationship between childhood sexual abuse, self-harm repetition and suicidal intent: mixed-methods study
Source: BJPsych Open. 2021 Jul 8;7(4):e125. doi: 10.1192/bjo.2021.962 (PMC8281309; doi:10.1192/bjo.2021.962)
Supplement: Supplementary file 1 [file S2056472421009625sup001.zip › S2056472421009625sup008.docx]

## Appendix 3. Reasons for not taking part in in-depth interview study

| Reasons for not taking part (n=152) | | | | |
| --- | --- | --- | --- | --- |
|  | Cork University Hospital | Mercy | Limerick | Total |
| No contact details | 0 | 10 | 6 | 16 |
| Never answered phone/phone disconnected | 5 | 28 | 14 | 47 |
| Engaged but stopped responding | 12 | 13 | 9 | 34 |
| Refused at first call | 3 | 16 | 5 | 24 |
| Not possible to approach (e.g. violent behaviour) | 5 | 15 | 11 | 31 |
| Total | 25 | 82 | 45 | 152 |
