# Supplementary material for: Investigating the relationship between childhood sexual abuse, self-harm repetition and suicidal intent: mixed-methods study
Source: BJPsych Open. 2021 Jul 8;7(4):e125. doi: 10.1192/bjo.2021.962 (PMC8281309; doi:10.1192/bjo.2021.962)
Supplement: Supplementary file 1 [file S2056472421009625sup001.zip › S2056472421009625sup007.docx]

## Appendix 2. Information collected for the in-depth interview study

|  | **Baseline interview items** |
| --- | --- |
| **Demographic information** | Date of birth  Gender  Nationality  Ethnicity  Religion  Marital status & children  Accommodation and living arrangements  Employment details  Level of education |
| **Self-harm act** | Description of method of self-harm act  Circumstances around self-harm act (objective and subjective) Beck Suicidal Intent Scale  Precipitants - Stressful and traumatic events (long checklist) |
| **History** | Recent symptoms of depression and mania  Family/ personal history  Expanded life events 12-item List of Threatening Experiences  History of non-fatal suicidal behaviour (expanded)  Suicidal behaviour by persons known to the deceased  Primary care history (short)  Psychiatric history (incl. views of service)  Physical health  Alcohol and drug abuse  Impulsivity 12-item Dysfunctional Impulsivity subscale of the Dickman Impuslvity Inventory  Coping 28-item Brief COPE  Self-efficacy 10 item General Self-Efficacy Scale  Social network 10-item version of the Duke Social Support Inventory  Hopelessness 4-item version of Beck Hopelessness Scale  Wellbeing 21 item version of the Depression Anxiety and Stress Scales |
